# Inequality as information: Wealth homophily facilitates the evolution of cooperation

**DOI:** 10.1038/s41598-018-30052-1

**Published:** 2018-08-02

**Authors:** Tim Johnson, Oleg Smirnov

**Affiliations:** 1 Atkinson Graduate School of Management, Willamette University, Oregon, 97301 USA; 2 Department of Political Science, Stony Brook University, New York, 11794 USA

## Abstract

Free-riding produces inequality in the prisoners’ dilemma: cooperators suffer costs that defectors avoid, thus putting them at a material disadvantage to their anti-social peers. This inequality, accordingly, conveys information about a social partner’s choices in past game play and raises the possibility that agents can use the aggregation of past payoffs—i.e. wealth—to identify a social partner who uses their same strategy. Building on these insights, we study a computational model in which agents can employ a strategy—when playing multiple one-shot prisoners’ dilemma games per generation—in which they view other agents’ summed payoffs from previous games, choose to enter a PD game with the agent whose summed payoffs most-closely approximate their own, and then always cooperate. Here we show that this strategy of wealth homophily—labelled COEQUALS (“CO-operate with EQUALS”)—can both invade an incumbent population of defectors and resist invasion. The strategy succeeds because wealth homophily leads agents to direct cooperation disproportionately toward others of their own type—a phenomenon known as “positive assortment”. These findings illuminate empirical evidence indicating that viewable inequality degrades cooperation and they show how a standard feature of evolutionary game models—viz. the aggregation of payoffs during a generation—can double as an information mechanism that facilitates positive assortment.

## Introduction

Cooperation ought to occur rarely. As captured by the prisoners’ dilemma model^1^, individuals can cooperate to generate a benefit, *b* > 0, at a personal cost, *c* > 0, yielding net benefits, *b* − *c* > 0, or they can free-ride on another’s cooperation to obtain *b* while leaving their social partners with the costs of cooperation, −*c*. These incentives should undercut cooperation, causing defectors to proliferate and regularly produce the Pareto-suboptimal outcome of mutual defection, 0.

Contrary to those expectations, cooperation often occurs^2,3^. Humans, for instance, cooperate to raise children^4^, maintain natural resources^5^, implement successful vaccine programs^6^, engage in warfare^7^, and conduct trade^8^—to name a few of the myriad examples of human cooperation^9,10^. Non-human primates cooperate when grooming^11^, child rearing^12^, forming coalitions or alliances^13^, and—under certain conditions—hunting^14^. Various bird species breed and help cooperatively e.g.^15^, and they cooperate in laboratory experiments when short-term payoffs are made less salient^16^. Insects cooperate extensively^17^ and some plant species cooperate via less-competitive root growth in the presence of clones^18^ or kin^19^.

To explain such forms of cooperation, theoretical research has identified mechanisms that support the evolution of cooperation^20^. One group of mechanisms consists of population structures^21^ that promote cooperation even when agents naively cooperate. Examples of these population structures include network arrangements^22–27^; cf.^28^ and clustering patterns that facilitate group selection^29,30^. Another category of mechanisms consists of behavioral programs that channel cooperation disproportionately to organisms of a cooperative persuasion^31–35^. Direct^31,34,35^ and indirect^33^ reciprocity—as well as reputation judgement^36^, choosiness^37^, and tolerance^38^—fit within this category^20,39^, as do resource allocation institutions that are attuned to contribution levels^40^. Other mechanisms promote cooperation by magnifying these methods: punishment^41–44^, for instance, leverages the efficacy of indirect reciprocity^45^, group selection^41^, and spatial structure^43^, just as conformism amplifies network reciprocity^46,47^. A final category of mechanisms involves assortment strategies that direct cooperation not merely to other cooperators, but toward agents that use their same strategy—that is, agents of the same genetic “type”^48,49^. Behavioral programs that detect kin and then target cooperation to those kin exemplify such assortment mechanisms^50^.

The strategy studied in this paper fits into the lattermost category of behavioral programs. It amounts to a variant of the strategy COEQUALS^49,51^. Studied in an environment in which agents played multiple one-shot PD games per generation in a well-mixed population, the original version of COEQUALS stipulated that agents compare the sum of their past payoffs with those of their social partner and cooperate with partners who possess summed payoffs equal to their own, defecting otherwise. This strategy flourishes in environments in which a correlation exists between the strategy agents implement and the sum total of payoffs they possess at any point in a generation. In such environments, total payoffs serve as a valid cue of the strategy an agent adopts; thus, cooperating with partners who possess equal payoffs from past play amounts to cooperating disproportionately with agents of one’s own type—i.e. positive assortment^9^.

By illuminating how the information content of wealth inequality can facilitate the evolution of cooperation, research on COEQUALS differs from other work that examines the relationship between wealth inequality and cooperation. Earlier research considered how wealth inequality could promote collective action by creating unilateral incentives for the provision of public goods^52,53^; recent work has extended that line of inquiry into a spatial, evolutionary context that more-precisely identifies the conditions in which resource heterogeneity will promote cooperation^54^. Further research has investigated the related possibility of limiting participation in public goods games to agents that possess a certain level of wealth, which effectively segregates cooperative types from other agents and yields network reciprocity^55^. This previous research provides compelling insights into the relationship between wealth inequality and cooperation, but it differs from both past work on COEQUALS and our present investigation. In this paper, we consider wealth inequality and cooperation in a framework that does not allow for resource complementarities^53^, spatial dynamics^54^, non-regular network structure (i.e. anything other than a fully connected graph)^55^, or the type of wealth-related institutional rules underpinning wealth-based selection^55^. Those features of previous research are fascinating and informative not only to research on cooperation, but to a wide range of biological questions e.g.^56^; however, our focus is different. We aim to test whether wealth homophily—as implemented via the COEQUALS strategy—can foster cooperation in a challenging setting that does not involve spatial or network structures that are known to expedite the evolution of cooperation.

Furthermore, this focus also distinguishes our investigation from past research that studies how unequal social influence shapes the evolution of cooperation in spatial or network contexts^57–59^. A foundational body of research in the study of spatial and network games indicates that inequality in strategy transmission—due to teaching^57^, social diversity^58^, or, generally, collective influence^59^—can bolster network-driven cooperation^27^ in the face of participation costs^60^. This past work reveals conditions in which clusters of cooperators in spatial or network proximity can drive ever-higher levels of cooperation in a population and it highlights the important role of extrinsic social inequality—namely, uneven success transmitting strategies—in that process. Our research differs from this prior work by studying a well-mixed population: all agents can potentially join games with each other. Unlike previous research, clusters of cooperators cannot form via spatial proximity or network links in our model because no relational ties persist across one-shot games or generations in our model. Instead, our model focuses on whether a strategy involving wealth homophily—namely, COEQUALS—can generate positive assortment in a well-mixed population that lacks spatial or network structures known to favor cooperation.

In this challenging environment, the original version of COEQUALS—which cooperates with partners possessing equal wealth and defects on others^49,51^—produces high levels of defection because it often finds itself trapped in mutually destructive interactions with free-riders. Such circumstances occur because the original variant of COEQUALS forces agents to disregard a partner’s wealth when joining a game, even though they base their choice of whether to cooperate or defect entirely on that information. It appears more plausible that information about wealth would be used throughout a social interaction. Accordingly, the variant of COEQUALS studied herein reflects this intuition: it selects social partners based on wealth equality and, then, *always* cooperates with those partners.

## Methods

We study COEQUALS and other strategies in a model involving a population of *N* agents who play *r* one-shot prisoners’ dilemma (PD) games per generation. In each PD game, mutual cooperation returns *b* − *c* > 0, while free-riding yields *b* and leaves cooperating game partners with −*c*; mutual defection results in 0. To add realism to the model and to test the robustness of COEQUALS, we include a “noise” parameter, *γ*, to vary the values of *b* and *c* in *each* PD game. Thus, the benefit from cooperation is *b*′ ~ *U*(*b* − *γ*, *b* + *γ*) and *c*′ ~ *U*(*c* − *γ*, *c* + *γ*) subject to the *b*′ > *c*′ constraint.

Agents adopt one of three strategies: *defector* (*D*), *cooperator* (*C*), or *COEQUALS* (*E*). Agents adopting defector choose to defect in all PD games, whereas agents using cooperator naively cooperate in all PD games. Agents adopting COEQUALS always cooperate, but they do so with individuals whose cumulative payoffs approximate their own. After *r* games in a generation, the payoff to player *i* is

1 $${\pi }_{i}^{r}=\sum _{x=1}^{r}\,{\pi }_{x}$$

where *π*_*x*_ is the payoff in the one-shot game *x* of a generation. An agent *i* implementing *COEQUALS* views the payoffs of some portion, *α*, of other agents in the population and *chooses* a partner from that sample with the smallest difference in wealth (if several agents have exactly the same wealth, then a partner is chosen randomly among them):

2 $${\rm{\Delta }}{\pi }_{i,j}^{r}=|{\pi }_{i}^{r}-{\pi }_{j}^{r}|.$$

Agents implementing defector or cooperator are *randomly matched* with their game partners from the set of available players—i.e., those who have yet to play in the current round. After all *r* one-shot games are played in a generation, differential reproduction takes place via the discrete replicator dynamics^61^. The proportion of a type *Z* in generation *g* + 1 is

3 $${P}_{g+1}^{Z}=(1-w){P}_{g}^{Z}+w({P}_{g}^{Z}\frac{{{\rm{\Pi }}}_{g}^{Z}}{{{\rm{\Pi }}}_{g}})$$

where *w* = [0, 1] is the intensity of selection parameter^24^, ${{\rm{\Pi }}}_{g}^{Z}$ is the average payoff to agents of type *Z*, and ∏_*g*_ is the average population payoff. Mutants emerge in the population via the rate, *mr*.

To gain broad insight into the model, we first study evolutionary dynamics in a large population under each possible value of *b*, setting all other parameters to their median values and displaying the results in phase diagrams^62^. Subsequently, we allow model parameters to vary randomly in a computer simulation that studies the proportion of each strategy in the population over the course of 400 generations, *g*. We replicate the simulation *n* = 5,000 times. Given our focus on the emergence and subsequent evolution of cooperation, the initial incumbent population in the computer simulation solely consists of defectors (${P}_{1}^{D}=1,\,{P}_{1}^{C}=0$, $\,{P}_{1}^{E}=0$) in all simulation runs. Cooperators and COEQUALS enter the population via mutation at the exogenously defined rate, *mr*, drawn from a uniform distribution ranging from 0.001 to 0.01. The cost of cooperation is fixed at *c* = 1; we vary the benefit of cooperation via draws from a discrete uniform distribution, *b* ~ *U*{2, 4}, and add noise to that value by randomly drawing the noise parameter from a continuous uniform distribution, *γ* ~ *U*(0, 0.5). For each run, we vary the number of agents, *N* ~ *U*{200, 400}. We also randomly vary the proportion, *α*, of agents in the population whose past payoffs a COEQUALS adopter can view, thus allowing those agents to view a segment of the population ranging from a sample size of 2 (the smallest sample size distinct from random partner selection) to *N* (the whole population). The number of one-shot PD games per generation is varied via draws from a discrete uniform distribution, *r* ~ *U*{2, 10}, and we also vary the intensity of selection, *w* ~ *U*(0.1, 1).

After studying a baseline model containing the above features, we test the robustness of COEQUALS by pitting it, serially, against two subtle variants of itself. First, we study the performance of COEQUALS when it faces a mimic that emulates its strategy, but aims to defect in the final one-shot game of a generation. Second, we augment our baseline model with a version of COEQUALS that defects with some probability in any game of a generation.

The first of these two variants reflects the intuition that if a COEQUALS mimic knows, with certainty, when the final game of a generation will occur, then it could invade a population of COEQUALS agents by defecting in the final game of a generation. We label this mimicry strategy “Opportunistic COEQUALS” because the strategy compromises its established pattern of activity to exploit a profitable opportunity at the end of a generation. However, if Opportunistic COEQUALS does not know when the final game of a generation will take place, its success is unclear: agents adopting the pure form of COEQUALS would be able to avoid adopters of Opportunistic COEQUALS once they defect, due to the inequality the latter agents would create by free-riding. To test this reasoning and gauge the robustness of COEQUALS to such a mimic, we implemented Opportunistic COEQUALS in our simulation by programming the strategy to randomly choose a one-shot game of a generation in which to start defecting and we randomly varied the number of one shot games in each generation as opposed to each simulation run. These changes resembled a scenario in which agents are uncertain about the number of games in a generation. All other features of the simulation, including its parameter values, remained the same as in the baseline simulation that lacked Opportunistic COEQUALS.

The second of the two variants seeks to test the robustness of COEQUALS while also illuminating the mechanism that makes COEQUALS successful—namely, using wealth homophily to facilitate positive assortment. This variant, which we label “Probabilistic COEQUALS,” defects with a certain probability in any one-shot PD game. On the one hand, it would seem that COEQUALS would suffer in the presence of a strategy that occasionally defects after entering PD games with agents holding the same wealth—after all, such a strategy could obtain the gains from free-riding. On the other hand, if wealth homophily generates positive assortment, then Probabilistic COEQUALS would be self-destructive because it would pair with its own adopters and occasionally exploit them, thereby foregoing an opportunity to advance its fitness via mutually beneficial cooperation. Furthermore, occasional free-riding would lead adopters of Probabilistic COEQUALS to possess cumulative wealth different from adopters of COEQUALS, thus allowing the latter agents to avoid the former, exploitative agents. Due to these conflicting possibilities, we add Probabilistic COEQUALS to our baseline simulation—keeping all other parameters the same—to test the robustness of the pure form of COEQUALS.

These robustness checks allow us to examine whether COEQUALS succeeds when facing variants of itself that possess seemingly advantageous features. Combined with the baseline model, which tests COEQUALS against conventional defection and cooperation strategies, the robustness checks examine whether wealth homophily can generate cooperation in a well-mixed, unstructured population playing one-shot PD games.

## Results

**Figure 1:**
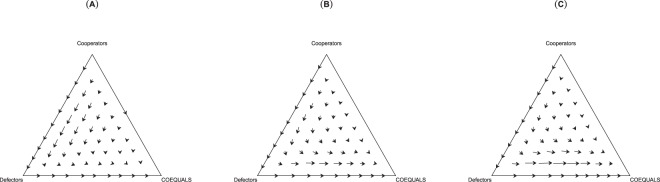
Evolutionary dynamics of COEQUALS, defectors, and cooperators. Figure 1 presents phase diagrams reporting evolutionary dynamics in the baseline model when *b* = 2 [Panel (A)], *b* = 3 [Panel (B)], and *b* = 4 [Panel (C)], and all other parameters—save for population size—are set to their median values. In Panels (A–C), simplex vertices represent states in which all agents in the population adopt the adjacently listed strategy. Points along a simplex’s edges signify states in which agents adopt some combination of two strategies, whereas points within the simplex represent states in which agents adopt some combination of three strategies. Arrows indicate the population’s trajectory and the length of an arrow’s tail indicates the speed of population change, with longer tails signifying faster population change. Panel (A) indicates that the population gravitates toward COEQUALS more rapidly when a substantial portion of the population consists of defectors. For instance, when *b* = 2, *P*^*D*^_*g*_ = 0.1, and *P*^*C*^_*g*_ = 0.8, the proportion of the population adopting COEQUALS grows by only 1% over a generation. However, when *b* = 2, *P*^*D*^_*g*_ = 0.8, and *P*^*C*^_*g*_ = 0.1, the proportion of the population adopting COEQUALS grows by roughly 15% over a generation. Indeed, even when *b* = 2, COEQUALS grows to fixation from a state of universal defection (*P*^*D*^_*g*_ = 1, *P*^*C*^_*g*_ = 0). As Panel (B and C) indicate, when the gains to cooperation increase, agents adopt COEQUALS at a faster rate and a smaller proportion of defectors in the population are needed for a rapid advance toward universal adoption. In comparison with the above analysis, Panel (B) indicates that when *b* = 3, the proportion of COEQUALS adopters grows by 15% over a generation when *P*^*D*^_*g*_ = 0.50 and the proportion of COEQUALS adopters grows by 33% when *P*^*D*^_*g*_ = 0.80 and *P*^*C*^_*g*_ = 0.1. When *b* = 4 [Panel (C)], the percent growth of COEQUALS adopters reaches 15% when *P*^*D*^_*g*_ = 0.39. At *P*^*D*^_*g*_ = 0.80 and *P*^*C*^_*g*_ = 0.10, COEQUALS grows by 53% over the course of a generation.

Findings from the model indicate selection for COEQUALS. In a large population with parameters set to their median values, evolutionary dynamics under each value of *b* show consistent movement of the population from states of widespread defection to universal adoption of COEQUALS (see Fig. 1). In the presence of cooperators, the population gravitates toward defection when a small proportion of agents adopt COEQUALS, but this trajectory turns toward greater adoption of COEQUALS as defectors become more common, thus eventually leading to the replacement of defectors with adopters of the COEQUALS strategy (Panels (A–C), Fig. 1). When *b* = 2, this shift toward adoption of COEQUALS occurs only after a substantial portion of the population has become defectors (Panel (A), Fig. 1), whereas it occurs with a smaller percent of defectors when *b* > 2 (Panels (A) and (B), Fig. 1). Overall, the phase diagrams suggest that COEQUALS performs better in a harsh environment brimming with defectors than a friendly population of cooperators.

In our computer simulation, we find that COEQUALS regularly dominates the population across runs. In 79% of all simulation runs, the majority of the population adopts COEQUALS by the final generation of the simulation (*g* = 400). Moreover, across runs, the average proportion of the population adopting COEQUALS at *g* = 400 equals 0.708 and the distribution of COEQUALS’ final share of the population rests on a median equaling 0.876.

COEQUALS also spreads across the population quickly. The majority of the population adopts COEQUALS, on average, by approximately the 44^th^ generation of a simulation run. The distribution of this statistic, furthermore, exhibits a rightward skew such that the median generation at which the majority of the population converts to COEQUALS is *g* = 23.

**Figure 2:**
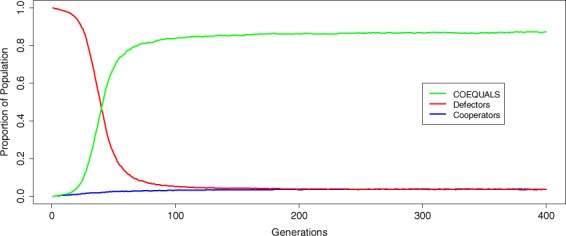
Growth and decline of strategies. From our 5,000 simulation runs, we gathered the distribution of each strategy’s proportion of the population in every generation; then, in Fig. 2, we traced the medians of these distributions from the first (*g* = 1) to the last generation (*g* = 400). The green line represents the median of COEQUALS’ distribution of population shares at each generation, while the red line conveys the corresponding information for the defector strategy and the blue line signifies that information for the cooperator strategy. The steep increase of the green line indicates the rapid spread of COEQUALS. The 23^rd^ generation is the median generation—across runs—by which COEQUALS spreads to over 50% of the population’s agents. From the 250^th^ to 400^th^ generation, the median percent of the population adopting COEQUALS approximately equals 87%, whereas defectors constitute a median percent less than 4% of the population during those generations. Furthermore, after the 250^th^ generation, the first-quartile of the distribution of COEQUALS’ percent of the population rests at approximately 59% and by the final generation the first-quartile of the distribution of COEQUALS’ population share increases to 64%, thus suggesting considerable selection for COEQUALS even in simulation runs in which the strategy grew less successfully.

COEQUALS’ proliferation coincides with a rapid decline in the proportion of defectors in the population. Figure 2 displays the divergent success of COEQUALS and defectors by tracing the median of each strategy’s distribution of population shares, in each generation, across the 5,000 runs of the simulation. By the 10th generation, the distribution of COEQUALS’ share of the population centers on a median value of approximately 0.01 and grows to 0.68 over the next 40 generations, continuing a gradual climb to 0.845 by the 110^th^ generation and stabilizing at a value of about 0.87 for the final 150 generations of the simulation (*g* = 250 to *g* = 400). During the same span, the distribution of defectors’ population shares at *g* = 10 balances on a median value of 0.98 before declining to 0.23 at *g* = 50 and settling between median values of 0.036 and 0.039 from the 200^th^ generation onward.

**Figure 3:**
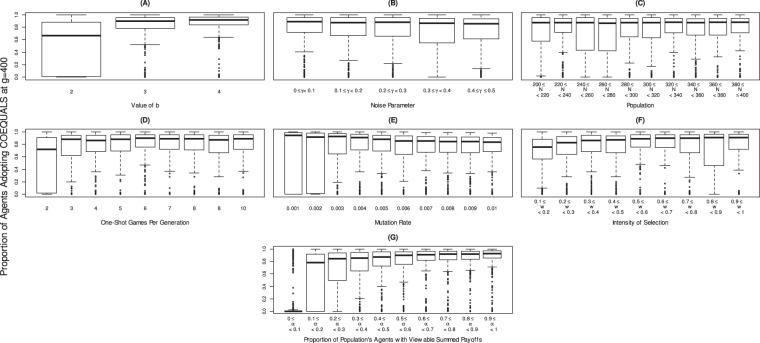
Proportion of population adopting COEQUALS in final generation by parameter value. Each panel displays the distributions of COEQUALS’ population shares at *g* = 400 across values of the parameter listed on the horizontal axis. Box plots in each panel display the median (bold center line), the upper/lower quartiles (upper/lower box edges), the non-outlier max./min. (uppermost/lowermost horizontal lines), and the outliers (hollow points) of the distributions, which are defined as observations that rest outside the interquartile range by a span greater than 1.5 times the interquartile range itself. Panel (A) shows that selection favors COEQUALS at larger values of *b*: the median of COEQUALS’ distribution of population shares increases noticeably when *b* > 2 and the lower tail of the distribution increases with every unit change of *b*. Varying the noise parameter has little effect on the spread of COEQUALS, however, as shown in Panel (B); across parameter ranges, the median of the distribution varies little and the shift of the distribution’s lower tail does not follow a discernable pattern. The same conclusions hold for variation across population sizes (Panel (C)) and the number of one-shot games per generation (Panel (D)). For both of those parameters, the distributions of COEQUALS’ population shares center on similar medians across all ranges of the parameter and the lower tails appear to vary arbitrarily. Panel (E) indicates that the distribution of COEQUALS’ population shares narrows with higher mutation rates, while the lower tails of the distributions increase in value. The substantive import of these changes remains relatively limited, however, as the interquartile ranges overlap substantially and the medians differ littler from each other, though they do exhibit a very slight decrease with higher mutation rates. Panel (F) indicates that the distribution of COEQUALS’ population shares varies little with the intensity of selection parameter, *w*. Variation in the parameter determining the proportion of other agents whose wealth a COEQUALS player can view (Panel (G)) shows the clearest trend. With every range involving higher values, both the median and the lower tail of COEQUALS’ distribution of population shares increases, albeit at a decreasing rate.

To understand why the population shifts from the defector strategy to COEQUALS, we studied the relationships between the final proportion of the population adopting COEQUALS and the model’s parameter values. As shown in Fig. 3, Panel (A), the distribution of COEQUALS’ final population share (*g* = 400) gathers closer to 1 when *b* > 2, but otherwise shows little variation across parameter values. Similarly, the median proportion of the population adopting COEQUALS at *g* = 400 varies little across values of, respectively, the noise parameter (Fig. 3, Panel (B)), the population size (Fig. 3, Panel (C)), the number of one-shot games per generation (Fig. 3, Panel (D)), the mutation rate (Fig. 3, Panel (E)), or the intensity of selection (Fig. 3, Panel (F)). For instance, across 0.1-unit spans of the intensity of selection parameter, *w*, the median of COEQUALS’ distribution of population shares at *g* = 400 remains relatively constant when *w* ≥ 0.3; moreover, the slight increase across categories from *w* = 0 to *w* < 0.3 is sufficiently modest that it would reside within the interquartile range of all distributions displayed in the diagram (Fig. 3, Panel (F)). Similar null patterns appear in the other panels and only the left tails of the distributions of COEQUALS’ final population shares vary noticeably across Panels (B) through (F) of Fig. 3; those left tails, however, vary in a haphazard manner, not in any recognizable pattern.

A pattern does appear in Panel (G) of Fig. 3, which displays the relationship between COEQUALS’ population shares at *g* = 400 and the proportion of the population’s wealth holdings that an adopter of COEQUALS can view. Panel (G) of Fig. 3 shows that when an adopter of COEQUALS can view the wealth of less than 10% of the population, COEQUALS rarely spreads to a majority of the population’s agents. However, when an adopter of COEQUALS can view the wealth of more than 10% of the population, but less than 20% of the population, the distribution of COEQUALS’ final population proportion shifts to the right and its median jumps to 0.79 (Fig. 3, Panel (G)). Moreover, with every 10 percentage-point increase in the pool of agents whose wealth can be viewed, the median and lower tail of the distribution of COEQUALS’ final population shares increases (Fig. 3, Panel (G)).

**Figure 4:**
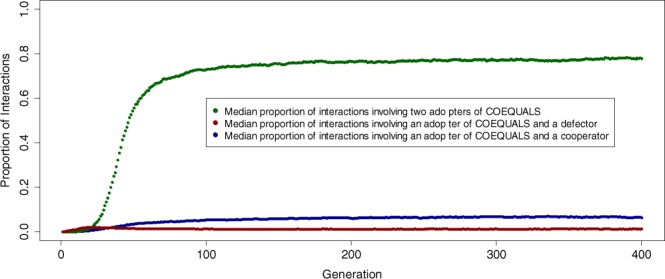
COEQUALS generates positive assortment. To understand whether COEQUALS generates positive assortment, we recorded the pair of strategies that agents used in every social interaction of our simulation; then, for every generation, we calculated the proportion of social interactions involving each possible pair of strategies. This created a distribution of 5,000 proportions indicating the share of social interactions involving each possible pair of strategies in each generation. Figure 3 plots the median of these distributions, by generation, for all interactions involving COEQUALS. It shows that, from the 21^st^ generation onward, the median proportion of interactions involving two adopters of COEQUALS (dark green points) exceeds the median proportion of interactions involving a COEQUALS adopter and a defector (dark red points). From the 10^th^ generation onward, the median proportion of interactions involving two adopters of COEQUALS exceeds the median proportion of interactions involving a COEQUALS adopter and a cooperator (dark blue points). Furthermore, within the first 50 generations, we find that the median proportion of interactions involving two COEQUALS players exceeds 50%, thus indicating that COEQUALS agents frequently begin to channel cooperation successfully to fellow COEQUALS adopters early in simulation runs. By the 200^th^ generation, the median proportion of interactions involving two COEQUALS adopters reached 0.76 and it remains roughly at this value for the remaining generations of the simulation.

Thus, a noticeable relationship exists between the success of COEQUALS and the ability of its adopters to view a wide segment of the population’s past payoffs. This relationship would be expected if COEQUALS proliferates due to positive assortment: expansion of the pool of agents with visible wealth holdings improves the ability of a COEQUALS agent to find an agent with equal wealth holdings and such agents have a higher chance of being another COEQUALS adopter. Indeed, we find evidence that COEQUALS succeeds due to positive assortment. Figure 4 shows that adopters of COEQUALS become ever more likely to channel cooperation to other COEQUALS agents—vis-à-vis agents employing other strategies—over the course of a simulation run.

**Figure 5:**
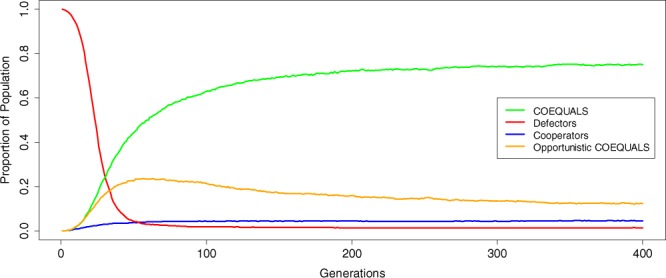
Robustness of COEQUALS to a variant that seeks to defect in the final game of a generation. The figure indicates the limited success of Opportunistic COEQUALS—a strategy that mimics COEQUALS, yet attempts to identify the final one-shot game in a generation and defect in it. Theoretically, COEQUALS appears vulnerable to this variant because it would gain the benefits of free-riding and impose the costs of cooperation on COEQUALS just prior to reproduction, thus acquiring a definitive fitness advantage. However, when the final game of a generation remains unknown, Opportunistic COEQUALS might defect prior to the final round of a generation, thus creating wealth inequality that gives COEQUALS agents the opportunity to avoid adopters of the variant and return to higher levels of fitness via mutually cooperating with other COEQUALS agents. The results in Fig. 4 dovetail with that possibility. The results show the median of the distribution of each strategy’s population share across every generation of the simulation. COEQUALS and the opportunistic variant hold the same median value until the 15^th^ generation of the simulation at which point the median of the distribution containing COEQUALS’ population shares increases relative to that of Opportunistic COEQUALS. Opportunistic COEQUALS, however, remains a non-trivial proportion of the population; the median of its distribution of population shares reaches a maximum value of 0.236 in the 57^th^ generation of the simulation and it remains at 0.123 at the end of the simulation. Despite this presence, the variant is eclipsed by the success of COEQUALS. The median value of COEQUALS’ distribution of population shares reaches its maximum value in the 346^th^ generation and hovers about this value through the end of the simulation, reaching it again in the 398^th^ generation. Indeed, from the 200^th^ generation onward, it continues to increase the median value of the distribution of its population shares, growing from approximately 0.72 at g = 200 to a final value of 0.75 at g = 400. Cooperators and defectors, on the contrary, constitute a trivial portion of the population throughout the simulation runs, with the latter strategy falling precipitously from its initial incumbent state.

The success of COEQUALS due to positive assortment raises the question of whether the strategy could be invaded by a mimic that employs the same behavioral program as COEQUALS, but defects in the final game of a generation. We find that the success of this mimic—labelled “Opportunistic COEQUALS”—is limited (Fig. 5). The distribution of Opportunistic COEQUALS’ proportion of the population at *g* = 400 centers on a value of 0.123 and in only 23% of simulation runs did the proportion of agents adopting Opportunistic COEQUALS in the final generation exceed 50%. To the contrary, the pure form of COEQUALS proliferates, albeit with attenuated growth, in the presence of Opportunistic COEQUALS. In the simulation, the majority of the population came to adopt COEQUALS in 71% of all runs. Furthermore, the average proportion of the population adopting COEQUALS at the end of a simulation run (*g* = 400) equals 0.63. This growth also occurs rapidly, with the majority of the population adopting COEQUALS, on average, by the 61^st^ generation of the simulation. Figure 5 provides further evidence of COEQUALS’ quick and sustained growth, even in the presence of Opportunistic COEQUALS.

**Figure 6:**
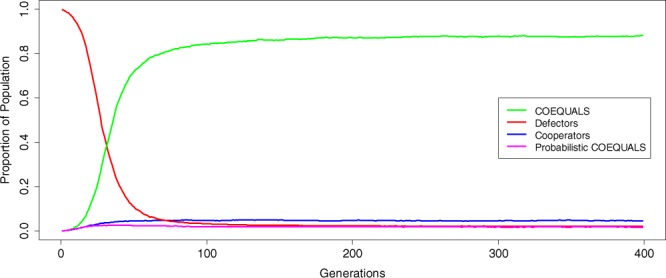
Robustness of COEQUALS to a variant that defects probabilistically with agents possessing the same wealth. Adopters of COEQUALS cooperate with certainty in every PD game they enter, thus raising questions about whether a variant of COEQUALS that defects with some probability in any game of a generation might outcompete the purely cooperative, baseline version of COEQUALS. Results presented in Fig. 6 show that this possibility does not materialize. The figure presents the median of the distribution of population shares of each strategy in every generation of the simulation. The results show selection against a variant of COEQUALS that probabilistically defects. Across generations, the median of the variant’s population shares reaches a maximum value of 0.025 and it dwindles beneath that value in the remaining generations, resulting in a final value of 0.016 in *g* = 400. To the contrary, the distribution of population shares for the purely cooperative form of COEQUALS centers on a median value that grows across generations. By the 200^th^ generation, the median of COEQUALS’ distribution of population shares rests on a value of 0.872, which grows to its maximum value of 0.881 by the final round of the simulation. The corresponding metrics for defectors and cooperators remain at low values over those same generations, thus suggesting that the conventional form of COEQUALS remains robust to all strategies in the presence of a variant of itself that probabilistically defects. This result is consistent with the idea that COEQUALS uses wealth holdings to generate positive assortment: in such conditions, defecting on others with equal wealth holdings reduces the overall fitness of the strategy and leads to missed opportunities for mutual cooperation.

The pure form of COEQUALS also outcompetes Probabilistic COEQUALS—a variant that enters games with its material equals, but chooses to defect with some probability. In simulation runs in which we add Probabilistic COEQUALS, the pure form of COEQUALS spreads widely such that the distribution of COEQUALS’ population shares in the final generation of the simulation centers on a median value of 0.881 (Fig. 6). Indeed, over half of all simulation runs result in over 85% of the population adopting COEQUALS by the 200^th^ generation. Probabilistic COEQUALS, on the other hand, struggles across simulation runs; the distribution of Probabilistic COEQUALS’ share of the population never achieves a median value, in any generation, above 0.025 and it declines to a final value of 0.016 in *g* = 400. Corresponding metrics for defectors and cooperators also remain at low values even with Probabilistic COEQUALS added to the simulation, thus suggesting that the conventional form of COEQUALS remains robust to a variant of itself that probabilistically defects. These results make sense in light of evidence that wealth homophily generates positive assortment. Given such evidence, adopters of Probabilistic COEQUALS occasionally exploit others who adopt their same strategy and, in so doing, they miss opportunities for mutually beneficial cooperation.

## Discussion

Together, these findings indicate a new mechanism for understanding the evolution of cooperation: wealth homophily. Notably, wealth homophily uses a central component of evolutionary game theory models—an agent’s summed payoffs—as an informational cue^51^. Evolutionary game models use the aggregation of payoffs to determine fitness, thus the mechanism we present here differs from other catalysts of cooperation—such as image scores^33^ or altruistic punishment^63,64^—which require the addition of new technologies to the basic PD model.

Furthermore, the findings of this paper illuminate past empirical research. Experimental research has shown that material inequality degrades cooperation when visible^65^. Our findings provide an explanation for why that is the case. Learning that others hold more or less wealth signals the presence of defectors in a community; thus, absent the option to choose partners, conditionally cooperative individuals who observe inequality would choose to defect to avoid being victimized by free-riding. Furthermore, research shows that humans show warmth toward equals, while exhibiting negative emotions and attitudes toward individuals who are either economically superior or inferior to themselves^66^. The current findings offer an ultimate explanation for these proximate dispositions: feeling positive emotions toward equals and negative emotions toward non-equals might facilitate the formation of cooperative relationships with others who employ a strategy of wealth homophily.

This link between equality and cooperation also points in the direction of offering an ultimate explanation for the relationship between individuals’ attitudes toward inequality and unfairness^67^. In our model, agents use inequality as a sign that individuals have engaged in a form of unfairness—namely, free-riding. Yet, individuals might also adapt their behavior to environments in which material well-being and past unfairness are uncorrelated. Exploring such possibilities and their relation to concerns of rank^68^ is a next step in this line of research.

The present research, however, simply shows new ways that inequality can inform human behavior. Whereas past research indicates that material inequality can inform risk-taking^69^ and expectations about economic opportunities^70^, we show that wealth inequality can reveal whether social partners employ one’s same strategy and, thus, whether one ought to join a cooperative relationship with them.
